# Variability in total serum IgE over 1 year in severe asthmatics

**DOI:** 10.1186/s13223-019-0331-8

**Published:** 2019-03-29

**Authors:** Renaud Louis, Charles Pilette, Olivier Michel, Alain Michils, Guy Brusselle, Antoine Poskin, Jan Van Schoor, Kris Denhaerynck, Stefaan Vancayzeele, Ivo Abraham, Sandra Gurdain

**Affiliations:** 1 Service de Pneumologie-Allergologie, CHU Sart Tilman B35, 4000 Liege, Belgium; 2 Cliniques Universitaires Saint-Luc, Avenue Hippocrate 10, 1200 Brussels, Belgium; 3 CHU Brugmann, Place A.Van Gehuchten 4, 1020 Brussels, Belgium; 4 CUB Hôpital Erasme, Route de Lennik 808, 1070 Brussels, Belgium; 5 UZ Gent, Corneel Heymanslaan 10, 9000 Ghent, Belgium; 6 Novartis Pharma, Medialaan 40, 1800 Vilvoorde, Belgium; 7 Matrix45, LLC, 6159 West Sunset Road, Tucson, AZ 85743 USA; 8 University of Basel, Basel, Switzerland

**Keywords:** Asthma, IgE, Variability

## Abstract

**Background:**

Immunoglobulin E (IgE) is the treatment target of omalizumab, a monoclonal antibody indicated in the treatment of severe allergic asthma. Long-term variability of serum total IgE (sIgE_tot_) in asthmatics remains poorly documented.

**Methods:**

In this prospective study, sIgE_tot_ levels were measured over 1 year at 7 time points in 41 severe asthmatics treated with high-dose of inhaled corticosteroids and long-acting β_2_ agonists. 33 patients were atopic based on at least one positive RAST to common aeroallergens. Patients were divided into three groups according to their baseline sIgE_tot_ level: low (< 76 IU/mL; n = 10), intermediate (76–700 IU/mL; n = 20) or high (> 700 IU/mL; n = 11). Patients also completed the six-item Juniper Asthma Control Questionnaire (ACQ_6_). The sIgE_tot_ variability and factors predictive for this variability were studied, as well as ACQ_6_ outcomes.

**Results:**

The variation in sIgE_tot_ level was mostly the consequence of between patient-variability, which represented 96%, 71% and 96% of the total variability in the low, intermediate and high sIgE_tot_ subgroups, respectively. The residual within-patient variability was therefore limited. In 10/41 patients, sIgE_tot_ levels increased or decreased, for at least one visit, beyond the predefined range of the subgroups to which they were assigned (< 76 IU/mL; 76–700 IU/mL; > 700 IU/mL). There was a significant but weak correlation between sIgE_tot_ and ACQ_6_ score over all time points (r = 0.15, p = 0.02), but sIgE_tot_ failed to associate with severe exacerbation. sIgE_tot_ decreased by 3% with any additional year of age for the whole group (p = 0.01) and increased by 5% per one unit of allergen exposure score in atopic patients (p = 0.002).

**Conclusion:**

In severe asthmatics, limited within-patient variability of sIgE_tot_ levels was observed over 1 year as opposed to marked between-subject variability. sIgE_tot_ decreases with age. Variation in sIgE_tot_ weakly associates with asthma control but not with exacerbation.

## Introduction

Immunoglobulin E (IgE) is an antibody associated with hypersensitivity and allergic reactions. IgE mainly binds on the high-affinity IgE receptor (FcεRI) on mast cells and basophils. Upon FcεRI cross-linking following allergen exposure, inflammatory mediators including histamine, leukotrienes and pro-inflammatory cytokines are released [1]. However, in murine models, the binding of IgE itself on mast cell surface may already favour cell survival and cytokine release irrespective of the presence of any allergen [2, 3]. Although direct evidence for this is still lacking in humans, it has been reported that asthmatics with high sputum IgE levels have also raised sputum levels of TNFα and Interleukin-6, two cytokines released from mast cells [4]. Therefore, IgE might possibly be considered as an inflammatory mediator independent of atopic status and deserves to be monitored.

In a study of a community population with measurements at two time points over an 8-year period, the level of serum total IgE (sIgE_tot_) tended to be stable in subjects above the age of 35 years while levels decreased in children and young adults. Atopic subjects tended to show a decrease with age even after the age of 35 years [5]. Further, a longitudinal study spanning a period of up to 20 years also showed that smoking opposed the natural decline in IgE and synergized with atopy to result in increased levels in subjects over the age of 50 years [6]. However, variation in sIgE_tot_ beyond two or three time points in the same subject has not been studied extensively.

Omalizumab, a monoclonal antibody that binds serum IgE, has become part of asthma treatment for the severe spectrum of the disease in which patients remain uncontrolled despite a combination of high dose inhaled corticosteroids (ICS) with long-acting β_2_ agonist (LABA). The drug has been validated in asthmatics whose serum IgE ranges from 30 to 700 IU/mL but most of the effect in terms of reduction of exacerbation was confined to patients with serum IgE ranging from 76 to 700 IU/mL [7]. A retrospective chart review of severe asthmatics with two to four measurements of sIgE_tot_ over an average period of 2 years showed some clinically significant variability in sIgE_tot_ affecting candidacy and dosing of omalizumab [8]. A recent prospective study conducted with 17 moderate-to-severe asthma patients with 6 serial measurements confirmed this variability in sIgE_tot_ over 1 year [9].

To better understand sIgE_tot_ variation and factors influencing this variation, we conducted a 12-month prospective study of sIgE_tot_ levels in severe asthmatics treated with high-dose ICS and LABA. Furthermore, we also examined how fluctuation in IgE may relate to day-to-day environmental exposure, asthma control and exacerbations.

## Methods

### Patient characteristics

Patients enrolled in the study had an established diagnosis of severe asthma as defined by the ERS/ATS consensus statement [10], aged ≥ 18 years, and currently treated with high-dose ICS (daily dose of fluticasone propionate > 500 µg or equivalent) and a LABA. Patients were excluded from the study if: (i) they were being or had been treated with omalizumab (Xolair^®^, Novartis, Basel, Switzerland), unless treatment was stopped at least 1 year before inclusion; (ii) they were undergoing immunotherapy; and/or (iii) used an investigational drug at the time of enrollment or within 30 days prior to enrollment in the study.

### Study design

This was a prospective, multicenter study to assess variability of sIgE_tot_ levels in severe asthmatics conducted in 5 Belgian centers. The 12-month variability in sIgE_tot_ levels in the total population was assessed and the proportion of the total variability explained by within- and between-patient variability was calculated (primary objective). In accordance with reimbursement criteria in Belgium, patients were stratified post hoc into three groups on the basis of baseline sIgE_tot_: < 76 IU/mL (low IgE), 76–700 IU/mL (intermediate IgE), and > 700 IU/mL (high IgE). These three groups were selected based on the study of Bousquet et al., which demonstrated that omalizumab reduces the annualized rate of asthma exacerbations more in patients with sIgE_tot_ ≥ 76 IU/mL [7]; and based on the upper limit of serum IgE inclusion criteria of the INNOVATE registration study (< 700 IU/mL) [11]. These two studies are currently used as the basis for the reimbursement of omalizumab in Belgium. An additional objective of the study was to evaluate demographic, environmental, and treatment factors that may influence sIgE_tot_ variation and whether sIgE_tot_ relates to asthma control and asthma exacerbation.

At baseline patients completed the six-item Juniper Asthma Control Questionnaire (ACQ_6_) [12]; blood was sampled to measure sIgE_tot_ levels; and atopic status of the patient was evaluated with a radio allergosorbent test (RAST) directed towards common allergens: grass mix, birch, mould mix, house dust mite (*Dermatophagoides pteronyssinus*), and cat and dog dander. Patients were considered atopic if RAST levels to any allergen exceeded 0.35 kU/L. Additionally, an environmental questionnaire was completed which enquired about the frequency of patients’ exposure (1: “once, more than 1 month ago”; 2: “once, more than 1 week ago”; 3: “once, within the last week”; 4: “occasionally”; 5: “frequently” or 6: “constantly”) to house dusts, pollens, molds, cats and dogs, smoke, humidifier/vaporizer, and carpeting.

Subsequently, 6 visits were planned every 2 months for a follow-up period of 12 months. At each visit, sIgE_tot_ was measured, the ACQ_6_ and environmental questionnaires were completed, and the treatment prescribed by the physician was registered. Treatment prescription, continuation, or discontinuation was per the physician’s best clinical judgment and in accordance with the Belgian reimbursement criteria. Also, to allow for the monitoring of exacerbations during the study, patients had to report if and how many times they had an exacerbation since the last visit. The presence of an exacerbation in the week preceding the visit was recorded specifically. A severe exacerbation was defined as an exacerbation requiring a treatment containing systemic corticosteroids for at least three consecutive days and/or an emergency room visit and/or a hospitalization.

This study was approved by the ethical committee of the CHU Liege under approval number B70720096731. All patients had to provide an informed consent before entering the study.

### Statistical analysis

Descriptive statistics were calculated using frequencies, percentages, means, standard deviations, medians and ranges as appropriate. Comparisons in baseline characteristics between the three subgroups (low, intermediate, high sIgE_tot_) were tested using ANOVA with Tukey post hoc test comparison for age and a general linear model with contrasts for other (categorical) variables. Random-intercepts regression analysis was used to model log-transformed sIgE_tot_, with ‘patient’ as the random variable. The model allowed us to calculate intra-cluster correlations to determine how much of the variability observed in sIgE_tot_ levels over time could be attributed to variability between individual patients, compared to variability within each patient. It also allowed us to test predictors of sIgE_tot_ (exposure to allergens, active/passive exposure to tobacco smoke, change in asthma treatment, treatment with maintenance systemic corticosteroids, and ACQ_6_ score). Modeling of exacerbations (absent/present) was performed by logistic regression analysis, using generalized estimating equations to account for repeated measurements over time. This analysis was used to assess influence of treatment changes and time of year. The correlation between sIgE_tot_ and ACQ_6_ score at baseline was evaluated with a Spearman correlation. As this was a real-life study, data were collected from routine clinical practice and there may be missing data. Missing values were not imputed in the descriptive and statistical analyses. p values < 0.05 were considered statistically significant.

## Results

### Patient characteristics

In total, 41 patients were recruited. The median sIgE_tot_ level at baseline in the entire study group was 248 IU/mL (range 17–7620 IU/mL). Mean ACQ_6_ score was 1.5 ± 0.87. Only 14/39 patients (35%; 2 responses unknown) had asthma GINA-defined as controlled at baseline. Of these 41 patients, 10 (27%) were included in the low sIgE_tot_ subgroup (≤ 76 IU/mL; median baseline sIgE_tot_ = 43.5 IU/mL, range 17–71 IU/mL); 20 patients (49%) were included in the intermediate sIgE_tot_ subgroup (76–700 IU/mL; median baseline sIgE_tot_ level = 247.0 IU/mL (range 86–667 IU/mL); and 11 patients (24%) were included in the high sIgE_tot_ subgroup (> 700 IU/mL; median baseline sIgE_tot_ level = 1332.0 IU/mL, range 729–7620 IU/mL). Atopy, defined as a positive RAST to at least one common aeroallergen, was present in 33/41 subjects (80%). The baseline characteristics by subgroup are given in Table 1.

**Table 1:** Baseline characteristics stratified by sIgEtot group (low, intermediate, high)

Baseline characteristics (N = 41)	Low IgE group (< 76 IU/mL) (N = 10)	Intermediate IgE group (76–700 IU/mL) (N = 20)	High IgE group (> 700 IU/mL) (N = 11)	*p* value**	Difference specification***
Age (years)					
Mean (± SD)	57.7 ± 15.7	49.5 ± 14.7	40.4 ± 13.4	0.03*	1 vs 3
Gender					
Male	5 (29%)	8 (47%)	4 (24%)	0.84	
Female	5 (21%)	12 (50%)	7 (29%)		
Race					
Caucasian	10 (28%)	19 (53%)	7 (19%)	0.03*	1 vs 3; 2 vs 3
Other	0 (0%)	1 (20%)	4 (80%)		
Smoking status					
Never smoked	6 (26%)	12 (52%)	5 (22%)	0.27	
Former smoker	3 (25%)	7 (58%)	2 (17%)		
Current smoker	1 (17%)	1 (17%)	4 (66%)		
BMI (kg/m^2^)					
< 30	9 (25%)	17 (47%)	10 (28%)	0.79	
≥ 30	1 (25%)	3 (75%)	0 (0%)		
Medical history					
Allergic rhinitis	2 (9%)	13 (56%)	8 (35%)	0.02*	1 vs 3; 1 vs 2
Atopic dermatitis	3 (17%)	11 (61%)	4 (22%)	0.33	
GERD	4 (44%)	4 (44%)	1 (11%)	0.23	
Nasal polyps	2 (25%)	3 (38%)	3 (38%)	0.59	
Residence					
City	4 (18%)	10 (45%)	8 (36%)	0.51	
Suburbs	4 (40%)	5 (50%)	1 (10%)		
Rural/country	2 (22%)	5 (56%)	2 (22%)		
GINA level of asthma control					
Controlled	3 (21%)	8 (57%)	3 (21%)	0.39	
Partly controlled	2 (18%)	7 (64%)	2 (18%)		
Uncontrolled	5 (33%)	4 (27%)	6 (40%)		
Positive RAST testing					
*D. pteronyssinus*	4 (18%)	11 (50%)	7 (32%)	0.65	
Grass mix	4 (22%)	6 (33%)	8 (44%)	0.07	2 vs 3
Cat dander	2 (12%)	10 (59%)	5 (29%)	0.41	
Dog dander	2 (12%)	7 (44%)	7 (44%)	0.16	
Birch	0 (0%)	5 (42%)	7 (58%)	0.005*	1 vs 3; 2 vs 3; 2 vs 3
Mold	1 (8%)	5 (42%)	6 (50%)	0.1	
Treatment					
ICS/LABA	10 (24%)	20 (49%)	11 (27%)	–	
SABA	5 (18%)	18 (64%)	5 (18%)	0.02*	1 vs 2; 2 vs 3
LTRA	2 (10%)	12 (60%)	6 (30%)	0.11	
OCS	1 (13%)	3 (37%)	4 (50%)	0.34	
SABA/SAAC	1 (33%)	1 (33%)	1 (33%)	1	
LABA	0 (0%)	1 (100%)	0 (0%)	1	
Asthma status					
% Predicted FEV1—mean (SD)	72.3 ± 28.6	86.6 ± 18.6	80.2 ± 12.2	0.20	
ACQ total score—median (range)	12.0 (2.0–14.0)	8.0 (5.0–13.0)	8.0 (4.0–13.0)	0.77	
Exhaled nitric oxide—median (range)	15.6 (7.1–46.7)	20.8 (9.6–154.0)	120.9 (5.7–236.0)	0.82	

BMI, Body Mass Index; *D. pteronyssinus*, *Dermatophagoides pteronyssinus*; FEV1, forced expiratory volume in 1 s; GERD, gastroesophageal reflux disease; ICS, inhaled corticosteroids; ICS/LABA, inhaled corticosteroids/long-acting β_2_ agonists; LABA, long-acting β_2_ agonists; LTRA, leukotriene receptor antagonist; OCS, oral corticosteroids; RAST, radio-allergo sorbent test; SD, standard deviation; SABA, short-acting β_2_ agonists; SABA/SAAC, short-acting anticholinergic

* p-value < 0.05 were considered statistically significant; ** Regression analysis for age/Fisher’s exact for other (categorical) variables; *** Tukey test for age/general linear model with contrasts for other (categorical) variables, the column indicates between which groups were observed the statistical differences: 1 = low sIgE_tot_ subgroup, 2 = intermediate sIgE_tot_ subgroup and 3 = high sIgE_tot_ subgroup

### Variability in sIgE_tot_ over 12 months

The between-patient variability represented 93.4% of the total variability for the entire sample, and 96%, 71% and 96% in the low, intermediate, and high sIgE_tot_ subgroups, respectively. Therefore, within-patient variability represented 4%, 29% and 4% of the total variability in the low, intermediate and high sIgE_tot_ subgroups, respectively. The within-patient variability was significantly higher in the intermediate sIgE_tot_ group than in the other groups (p < 0.0001) (Table 2).

**Table 2:** Intra-cluster correlation (ICC) of serum total IgE (sIgE_tot_) evaluated over the 12-month study period in three strata of asthmatic patients based on sIgE_tot_ at baseline: < 76 IU/mL (n = 10); 76–700 IU/mL (n = 20) and > 700 IU/mL (n = 11)

	ICC (95% CI) (*)	1-ICC (95% CI)	p-value (**)
< 76 IU/mL	96 (93–98) %	4 (2–7) %	
76–700 IU/mL	71 (62–82) %	29 (18–38) %	< 0.0001
> 700 IU/mL	96 (94–99) %	4 (1–6) %	

CI, confidence interval

* Between-patient variability/(between- + within-patient variability)** likelihood ratio test comparing within-patient variabilities

**Fig. 1:**
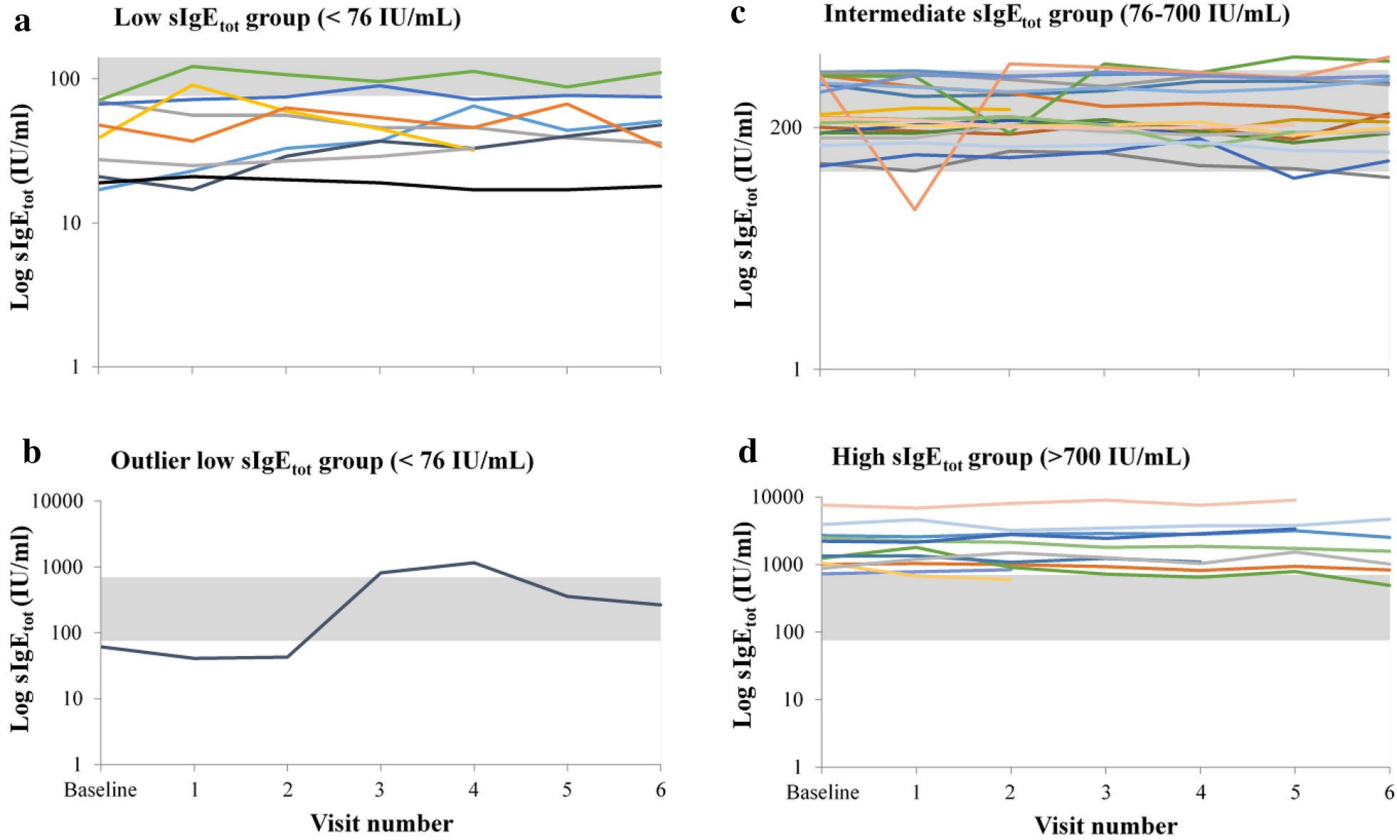
The 12-month variability in sIgE_tot_ in severe asthmatic patients stratified by sIgE_tot_ at baseline (low, intermediate and high). The 12-month variability in sIgE_tot_ was evaluated in 41 patients divided post hoc in three strata based on sIgE_tot_ at baseline: < 76 IU/mL (n = 10) (**a**); 76–700 IU/mL (n = 20) (**c**) and > 700 IU/mL (n = 11) (**d**). One outliner patient from the low sIgE_tot_ group is shown separately (**b**). The gray zone indicates the 76–700 IU/mL region (intermediate group) to allow for the identification of patients whose sIgE_tot_ reached the level of a different group during the study period

Detailed individual trajectories over time by baseline IgE strata are shown in Fig. 1, panels a, c, and d. Ten of the 41 patients (24%) reached values beyond the limit of their own baseline sIgE_tot_ subgroup during at least one of the follow-up visits. Of these 10 patients, 4 were in the low, 4 in the intermediate, and 2 in the high sIgE_tot_ subgroup, therefore representing 40% of the patients in the low sIgE_tot_ subgroup, 20% of the patients in the intermediate sIgE_tot_ subgroup and 18% of the patients in the high sIgE_tot_, respectively.

Two patients had sIgE_tot_ levels in the three categories over the 1 year period of observation. One patient initially in the low sIgE group had a rise in IgE above 700 IU/mL for at least 2 consecutive measurements before returning to intermediate levels at the two last visits (Fig. 1b). This patient was subsequently found to have developed a parasitic infection.

The relevant clinical data for this patient are presented in Table 3. The patient did not have any positive RAST results at baseline and was therefore considered non-atopic.

**Table 3:** Clinical data for the outliner patient belonging to the low serum total IgE (sIgEtot) subgroup (see Fig. 1b)

Visit	Baseline	1	2	3	4	5	6
sIgE_tot_ (IU/mL)	61.3	41	43	818	1159	357	266
FEV1 predicted (%)	49		61	64	52	59	65
Total ACQ score	13	19	14	25	19	15	16
FENO (ppm)	7.1		4.8	18.6			28.1
Asthma exacerbation	x	x	x	x	x		
Asthma exacerbation requiring OCS (if yes dosis mg)				32	16		
Parasitic infection				x			
Budesonide/formoterol (µg/days)	1200/54	1200/54	800/36	1200/54	1200/54	1200/54	1200/54
OCS dosis (mg/days)	16	16	8	16	16	16	16
Beclamethasone/formoterol		200/18	200/18			200/18	200/18
Salbutamol	x			x	x		
Fenoterol/ipratropium			x				
Theophylline	x		x	x		x	x
Azythromycine	x	x	x				

FEV_1_, forced expiratory volume in 1 s; FENO, fractional exhaled nitric oxide; OCS, oral corticosteroids

### Relationship between variation in sIgE_tot_ and demographics, environmental exposure and treatment features

Age was a significant predictor of sIgE_tot_ levels over 12 months. sIgE_tot_ decreased by 3% with each additional year of age (p = 0.01). Allergen exposure for patients with a history of a positive RAST test was associated with change in sIgE_tot_. Exposure scores ranged from 0 to 12 and reflected the sum of the frequencies of exposure for the different allergens for which the patient had shown a positive RAST. A unit increase in the exposure score was associated with a 5% rise sIgE_tot_ (p = 0.002). No relationship was found between sIgE_tot_ and gender, smoke exposure, and asthma treatment.

### Relationship between sIgE_tot_ and lung function, asthma control, and exacerbation

There was no relationship between lung function (% predicted FEV1) and sIgE_tot_ variation at baseline or throughout the study period. The odds of severe asthma exacerbations the year prior to enrollment were not correlated significantly with baseline sIgE_tot_ in the total study group (OR = 0.95; p = 0.21), nor in the low (OR = 0.85; p = 0.95), intermediate (OR = 0.82; p = 0.42) or high subgroup (OR = 0.96; p = 0.31). Over the entire study period, there was no correlation between sIgE_tot_ and severe exacerbations since the previous visit nor with exacerbation that occurred the week before the visits. At baseline there was no relationship between sIgE and ACQ_6_ in the whole group (Spearman’s ρ = 0.01, p = 0.96) nor in any of the three groups. There was a significant but weak correlation between sIgE_tot_ and ACQ_6_ score across all time points (r = 0.15, p = 0.02).

### Influence of seasons on sIgE_tot_ asthma control

**Fig. 2:**
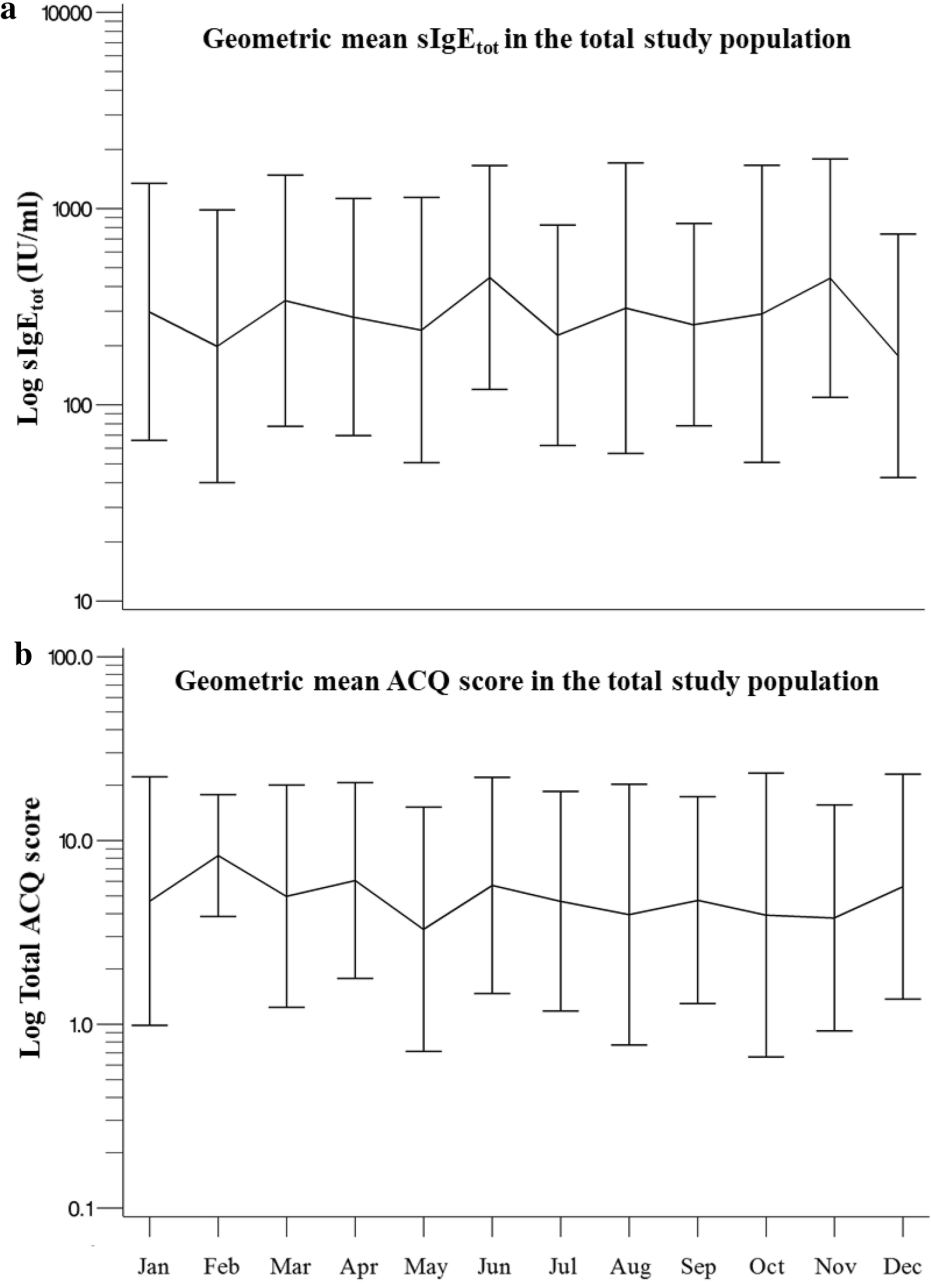
Variation in sIgE_tot_ and asthma control in severe asthmatic patients (n = 41) according to the seasons. Data from the 6 follow-up visits are shown according to the month of the visit (the baseline data are not included). In these graphics, the geometric mean sIgE_tot_ with standard deviation (SD) (**a**) and the geometric mean six-item Juniper Asthma Control Questionnaire (ACQ_6_) score with SD (**b**) are given

Figure 2 shows the variation in sIgE_tot_ (panel a) and asthma control (panel b) according to the seasons. In the total study group, there was no association between seasons (March–May, June–August; September–November; December–February) and sIgE_tot_ (p = 0.47) or ACQ_6_ score (p = 0.43).

## Discussion

There are limited data in the literature on the stability of IgE measurements over time in asthmatics. In the current study, we investigated variations in sIgE_tot_ in severe asthmatics over a 1 year period with repeated measurements every 2 months. The principal finding is that most of the variability sIgE_tot_ was due to between-patient variability whereas within-patient variability of sIgE_tot_ levels was rather limited. sIgE_tot_ was found to decrease with age.

The relative stability in sIgE_tot_ suggests that the mechanisms regulating IgE levels are sustained over time in most patients. Nonetheless, 24% of patients showed sIgE_tot_ level scores beyond the range of their own group for at least one visit. This was particularly the case in patients with baseline sIgE_tot_ < 76 IU/mL as 4 out of 10 patients had a measurement above 76 IU/mL in at least one subsequent blood sample.

Omalizumab is a biologic with proven efficacy to improve asthma quality of life and reduce asthma exacerbations in severe allergic asthmatics. The findings from our study suggest that regular monitoring of sIgE_tot_ in routine clinical practice may assist in optimizing treatment by aligning it with observed variations in sIgE_tot_ and thus avoid under-treatment with omalizumab. As the 40% of patients with sIgE_tot_ < 76 IU/mL at baseline but subsequent serum levels in the 76–700 IU/mL range underscore, without repeated blood sampling these patients might not have been initiated on omalizumab, which may have translated into a subsequent preventable need for corticosteroid therapy or into preventable exacerbations and hospitalizations. Likewise, the 20% of patients with sIgE_tot_ between 700 and 1000 IU/mL who subsequently showed serum levels below the 700 IU/mL threshold might not have been initiated on omalizumab without repeated testing. However, patients with very high serum IgE levels (> 2000 IU/mL) remain fairly stable in this “high” zone. Although between-patient variability was the major trend identified in our study, limited within-patient variability was still present. Therefore, regular monitoring of sIgE over 1 year results in an increase from 50 to 66% of patients falling into the range between 76 and 700 IU/mL. The limited patient variability resulted in the fact that two-third (27/41) of our patients treated with high doses ICS/LABA had a serum IgE values between 76 and 700 IU/mL after a 1 year period of observation (including 7 measurements for most of them), a figure clearly higher than 50% of patients as observed at baseline.

Despite the limited within–patient variability, one patient with baseline sIgE_tot_ < 76 IU/mL showed significant changes in serum levels over the observation period. This patient who was non-atopic, maintained low IgE levels (< 76 IU/mL) at the first two visits, after which there was a marked increase to 818 IU/mL at the third and 1159 IU/mL at the fourth visit, before declining at subsequent visits without, however, returning to low serum levels. This patient was a severe corticosteroid-dependent asthmatic in whom a parasitic infection was detected and treated accordingly. Remarkably, when the sIgE_tot_ increased sharply at visit 3, the patient’s ACQ_6_ scores rose from 2.3 to 4.2 without any change in FEV_1_ but with an augmentation of the OCS dose as treatment adjustment. This case points at the importance of considering possible etiologies when a sharp rise in IgE is detected. IgE levels are known to increase in the case of parasitic infection, namely helminth infections [13]; yet other causes of elevated IgE outside an atopic status should also be considered including infections by mycobacterium tuberculosis, Epstein–Barr virus, cytomegalovirus, malignancies, or chronic inflammatory/dysimmune disorders [14].

Though not powered for that purpose, our study also showed an inverse relationship between age and serum IgE, a finding we have recently reported in a large cross-sectional study in asthmatics [15] and which was previously established in longitudinal population studies [5, 6]. A decrease in sIgE_tot_ by 3% with every year increase in age was observed in the present study. Not unexpectedly, allergen exposure score was associated with a rise of sIgE_tot_ in sensitized subjects, supporting a role for allergen exposure in IgE synthesis in addition to its role in mast cell activation thereby confirming a recent study [9].

In contrast to what is known for eosinophils [16, 17], there are limited data on the relationship between sIgE_tot_ and asthma control and severity. A higher sIgE_tot_ level in severe asthmatics as compared to that in mild to moderate asthmatics has recently been reported in a large real-life cross-sectional study even if there was a considerable overlap between the groups [15]. In the same study, there was however no correlation between ACQ_7_ and serum IgE. In the study reported here, though not present at baseline, a weak but significant correlation was observed between sIgE_tot_ and ACQ_6_ score throughout the study period, which suggests that fluctuation in IgE over time in a subject may relate to day-to-day asthma symptoms. By contrast, no relationship was found between sIgE_tot_ and severe exacerbation rates. Recent data from SARP (Severe Asthma Research Program) have shown an inverse and surprising relationship between serum IgE and propensity to severe exacerbation in asthmatics [18].

Omalizumab convincingly reduces asthma exacerbation in clinical trial and routine practice in patients with serum IgE ranging from 30 to 700 IU/mL. The efficacy of omalizumab was shown when free circulating IgE was reduced by more than 95% and maintained at lower than 30 IU/mL [19]; however, sIgE_tot_ itself prior to treatment initiation does not predict the ability of the drug to prevent exacerbation [20]. Rather than considering the baseline IgE level, the IgE level at which omalizumab is able to maintain free circulating IgE may be more relevant in terms of reduction in exacerbations. It is also likely that airway (and not serum) IgE, which can be assessed in sputum [4, 21] and bronchial biopsies [22, 23], plays a more important role in the disease than circulating IgE though a convincing correlation was reported between serum and sputum IgE levels [4]. We might have found a stronger relationship between IgE and exacerbation if we had measured airway IgE rather than serum IgE in the present study.

The main limitation of our study is the limited number of patients included and followed up throughout the protocol. This may have resulted in a lack of association between sIgE and seasons in contrast to what was reported by others [9]. Moreover, we did not found a relationship between smoke exposure and serum IgE. Our negative finding does not preclude, however, any real effect of smoke exposure on serum IgE because of the limited number of current smoking asthmatics in our cohort. Future studies with larger sample sizes are needed to confirm our findings and explore associations with other possible determinants of IgE variation. Asthmatics in this study were selected on the basis of disease severity defined by the requirement of high-dose ICS combined with a LABA. Therefore, the IgE variability depicted here may not be applicable to a population of mild untreated asthmatics. As we did not have detailed history data regarding atopic dermatitis we could not assess the influence of the disease evolution on sIgE_tot_ over the study period; specifically, whether change in atopic dermatitis severity may have influenced change in serum IgE in our study. In our study the majority of patients with atopic dermatitis were in the intermediate IgE group.

We conclude that in severe asthmatics receiving high-dose ICS combined with a LABA, there was limited within-patient variability of sIgE_tot_ but significant between-patient variability. Yet 30% of the patients not initially eligible for receiving omalizumab based on a single measurement may actually benefit from repeated measurements to qualify for treatment.

## Abbreviations

ACQ: Asthma Control Questionnaire
ACQ_6_: six-item Juniper Asthma Control Questionnaire
BMI: Body Mass Index
*D. pteronyssinus*: *Dermatophagoides pteronyssinus*
FcεRI: high-affinity IgE receptor
FEV_1_: forced expiratory volume in 1 s
FENO: fractional exhaled nitric oxide
GERD: gastroesophageal reflux disease
ICC: intra-cluster correlation
ICS: inhaled corticosteroids
ICS/LABA: inhaled corticosteroids/long-acting β_2_ agonists
IgE: immunoglobulin E
LABA: long-acting β_2_ agonist
LTRA: leukotriene receptor antagonist
OCS: oral corticosteroids
OR: odds ratio
RAST: radio-allergo sorbent test
SABA: short-acting β_2_ agonists
SABA/SAAC: short-acting β_2_ agonists/short-acting anticholinergic
SARP: Severe Asthma Research Program
SD: standard deviation
sIgE_tot_: serum total IgE
